# Inter-species inference of gene set enrichment in lung epithelial cells from proteomic and large transcriptomic datasets

**DOI:** 10.1093/bioinformatics/btu569

**Published:** 2014-08-24

**Authors:** Sahand Hormoz, Gyan Bhanot, Michael Biehl, Erhan Bilal, Pablo Meyer, Raquel Norel, Kahn Rhrissorrakrai, Adel Dayarian

**Affiliations:** ^1^Kavli Institute for Theoretical Physics, Kohn Hall, University of California, Santa Barbara, CA 93106, USA, ^2^Department of Physics, ^3^Department of Molecular Biology and Biochemistry, Busch Campus, Rutgers University, Piscataway, NJ 08854, USA, ^4^Johann Bernoulli Institute for Mathematics and Computer Science, University of Groningen, 9700 AK Groningen, The Netherlands and ^5^IBM T.J. Watson Research Center, Computational Biology, Yorktown Heights, NY 10003, USA

## Abstract

**Motivation:** Translating findings in rodent models to human models has been a cornerstone of modern biology and drug development. However, in many cases, a naive ‘extrapolation’ between the two species has not succeeded. As a result, clinical trials of new drugs sometimes fail even after considerable success in the mouse or rat stage of development. In addition to *in vitro* studies, inter-species translation requires analytical tools that can predict the enriched gene sets in human cells under various stimuli from corresponding measurements in animals. Such tools can improve our understanding of the underlying biology and optimize the allocation of resources for drug development.

**Results:** We developed an algorithm to predict differential gene set enrichment as part of the sbv IMPROVER (systems biology verification in Industrial Methodology for Process Verification in Research) Species Translation Challenge, which focused on phosphoproteomic and transcriptomic measurements of normal human bronchial epithelial (NHBE) primary cells under various stimuli and corresponding measurements in rat (NRBE) primary cells. We find that gene sets exhibit a higher inter-species correlation compared with individual genes, and are potentially more suited for direct prediction. Furthermore, in contrast to a similar cross-species response in protein phosphorylation states 5 and 25 min after exposure to stimuli, gene set enrichment 6 h after exposure is significantly different in NHBE cells compared with NRBE cells. In spite of this difference, we were able to develop a robust algorithm to predict gene set activation in NHBE with high accuracy using simple analytical methods.

**Availability and implementation:** Implementation of all algorithms is available as source code (in Matlab) at *http://bhanot.biomaps.rutgers.edu/wiki/codes_SC3_Predicting_GeneSets.zip*, along with the relevant data used in the analysis. Gene sets, gene expression and protein phosphorylation data are available on request.

**Contact:** hormoz@kitp.ucsb.edu

## 1 INTRODUCTION

Although rodents diverged from primates around 75 million years ago, the common origin of eukaryotic multicellular organisms suggests that basic mechanisms in all complex life should be governed by similar pathways and principles. This simple observation has led to the successful use of *in vivo* rat and mouse models to understand biological mechanisms in humans. In particular, the initial phases of drug development rely heavily on knowledge gained from *in vitro* and *in vivo* experiments on model organisms, primarily from rat and mouse models. However, rats or mice often differ from humans in their response to stimuli (Hackam *et al.*, 2006; Pound *et al.*, 2004; Rice, 2012; van der Worp *et al.*, 2010). An open problem in the field is to understand these differences, especially those that affect mechanisms of pathway activation. There remains a continuing need to develop better analytical methods and modeling techniques to translate information from *in vitro* rat/mouse studies to accurate models of pathway activation in humans.

In addition to response differences at the tissue level, one also expects differences at the cellular level in rat and human cells subject to the same stimulus. Part of the difference in cellular response is probably because of changes in the promoter and gene sequence that alter cellular pathways. This would result in changes in expression levels, thresholds, reaction rates and activation times resulting in differences in the way a stimulus may activate a given pathway. For instance, mouse embryonic stem cells self-renew when the LIF/Stat-3 signaling pathway is activated, but human embryonic stem cells do not (Sato *et al.*, 2004); both cell types, however, respond similarly to Wnt activation (Sato *et al.*, 2004). Similarly, mouse and human leukocytes respond differently in terms of their gene expression to stimuli that induce inflammation (Mestas and Hughes, 2004; Seok *et al.*, 2013).

In general, one expects that there may be significant differences between species in the way single genes get activated. However, the regulation of collections of genes that act in concert (gene sets) to activate pathways, or respond to activation of a pathway, might be expected to be more similar (Subramanian *et al.*, 2005). As many cellular pathways are regulated by phosphorylation levels of specific families of proteins, one way forward is to develop methods to predict activation of pathways in human cells from measurements of protein phosphorylation and gene (or gene set) expression levels in rats under a variety of stimuli.

The sbv IMPROVER (systems biology verification in Industrial Methodology for Process Verification in Research) Challenges are an industry initiative focused on verifying the strengths and weaknesses of systems biology methods on a variety of biological problems by tapping the ‘Wisdom of Crowds’ (Meyer *et al.*, 2012). As a part of this goal, the organizers held the Species Translation Challenge (STC), a challenge specifically focused on translating biological responses observed in rat to human, given the same set of perturbations in comparable cell lines (Rhrissorrakrai *et al.*, 2015). This challenge allowed teams of researchers, including us, to compare the performance of different methods to translate results within and between species.

In the specific challenge described here, the so-called Inter-Species Pathway Perturbation Challenge (Sub-challenge 3 or SC3), we used phosphorylation level and gene expression data from normal rat bronchial epithelial (NRBE) and normal human bronchial epithelial (NHBE) cells to develop a predictive model of the activation of pathways/gene sets in humans from measurements in rats. The model was trained on a set of stimuli and tested on data from the same experimental design using different stimuli. In the training data, we were provided with measurements on both rat and human cells, whereas in the test data, we were provided only with measurements in rat. The challenge was to develop a model on the training data and use it to predict pathway activation in human cells using the rat test data. The results from each team were compared with a ‘gold standard’ dataset on human cells using the same stimuli. Our method, which we describe below, was judged to be the best performer in this sub-challenge.

**Fig. 1.**
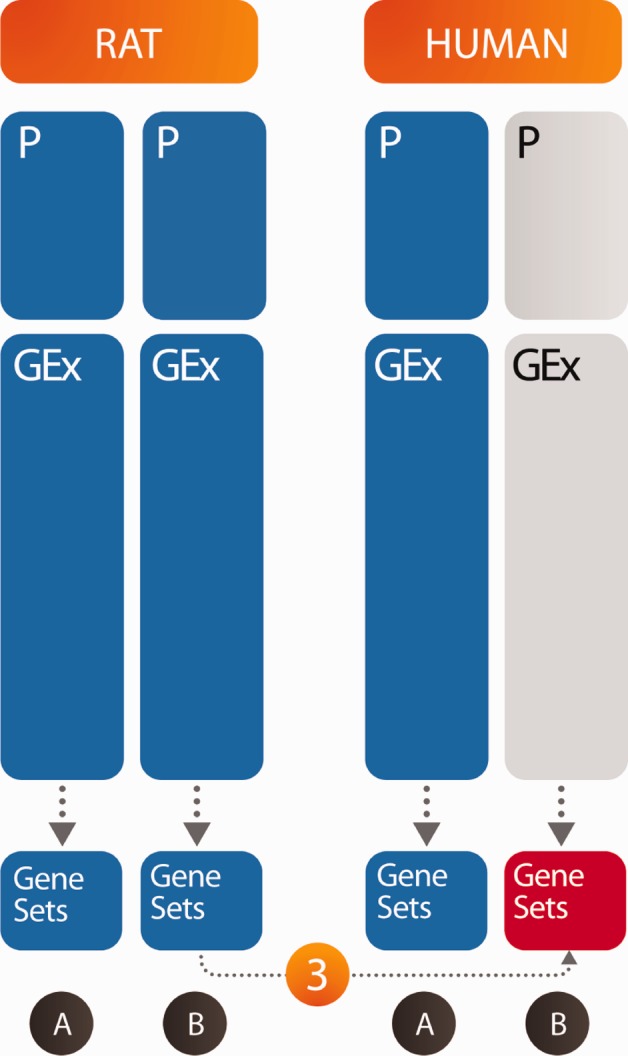
We predict gene set enrichment in human bronchial epithelial cells under a diverse set of stimuli (B) from measurements of gene set enrichment in rat cells under the same stimuli. We considered two distinct approaches: (i) a direct method where the algorithm was trained only on the gene set measurements (set A), and a direct prediction was made on the enrichment scores of set B. (ii) An indirect method where the gene expression levels were used for training and prediction. A GSEA was then used to infer the gene set enrichment scores. Blue boxes are the available data; red is to be predicted

The outline of the article is as follows. First, we describe how the raw data were processed to identify statistically significant signals. Next, we discuss inter-species correlations (human and rat) for both gene sets and gene expression level data. In principle, there are two possible strategies available to predict differential expression (enrichment) of human gene sets from the rat data (see Fig. 1): (i) one possibility is to use a direct method, where the human gene set enrichment scores are learned from those in rat training data (Rat A/Human A) and then applied to the rat test data gene set scores (Rat B) to predict human gene set scores; (ii) the second possibility is to use an indirect method, where human gene expression levels are learned from gene expression levels in rat in the training data and applied to the Rat B gene expression test data to get human expression predictions. Finally, the predicted Human B gene expression levels could be used in a gene set enrichment algorithm (GSEA) (Subramanian *et al.*, 2005) to predict human gene set scores. Although we attempted both methods, for our final prediction, we used only the direct method [method (i)]. Below, we outline our reasons for choosing the direct method followed by a detailed description of the prediction algorithm we used for our predictions.

## 2 METHODS

### 2.1 Data generation

Half of the human data generated for the STC was made available to the participants as training data (Poussin *et al.*, 2014), while the other half was kept hidden from participants and used as the gold standard to score participant predictions in the STCs SC3. The data consisted of phopshoproteomic, cytokine and gene expression data measured on normal bronchial epithelial primary cells from human and rat. These cells were exposed to 52 different compounds or to normal growth media for control (DME: Dulbecco’s modified Eagle’s medium) and then lysed at different time points depending on the type of measurement done. Phosphoproteins were measured at 5 and 25 min , gene expression was measured at 6 h and cytokines at 24 h after adding the stimulus (compound). The exposure of cells to each stimulus was performed in triplicate, and in four to six replicates for the DME control. The phopshoproteomic experiments were performed using Luminex xMAP technology and involved measuring the phosphorylation status of 16 proteins under the different conditions. Similarly, 22 cytokines were measured using the same platform.

The gene expression data were collected on Affymetrix HG-U133 Plus2 platform for human cells and Rat 230 2.0 for rat cells and processed using GCRMA (Wu *et al.*, 2004). For a more complete description of the data generation and a list of genes and gene sets, see Rhrissorrakrai *et al.*, 2015 in this issue or (Poussin *et al.*, 2014).

### 2.2 Processing gene expression data

Our primary goal in the processing of the gene expression data was to remove artifacts in the data and extract the statistical significance of the remaining signal. We were motivated by two broad characteristics of microarray data: (i) existence of outliers owing to various systematic errors that are not correctable using standard statistical tests; (ii) a saturation effect observed in the measurements, caused by a saturation of the fluorescence signal resulting from the specifics of hybridization and imaging techniques used in microarray technology. Although it is claimed in the conventional use of microarray technology that the non-linearity introduced in the signal by this saturation effect should not impact identification of differentially expressed genes, we believe that it distorts the true correlation between the gene expression signal and the level of phosphorylation of the corresponding proteins. We decided to correct the saturation effect by studying the properties of noise in the gene expression data.

First, we characterized the level of noise in the gene expression data. Our starting assumption is that the observed noise is because of the measurement process. Biological fluctuations in gene expression are averaged out because of the ensemble (many cell) nature of the measurement. However, there is still variation in expression level from multiple measurements on the same biological extract. The present dataset had few replicates, which creates a substantial problem in identifying true measurement noise and makes it difficult to pinpoint outlier values. The statistical uncertainty in the standard deviation estimated from three/four replicates is large. To get around these issues, we made the reasonable assumption that the measurement noise is only a function of the mean expression level and independent of the gene type. Although this ignores potential sequence-dependent hybridization effects, it has been shown to be true on average (Tu *et al.*, 2002), and it allows us to characterize the noise to a good accuracy.

**Fig. 2.**
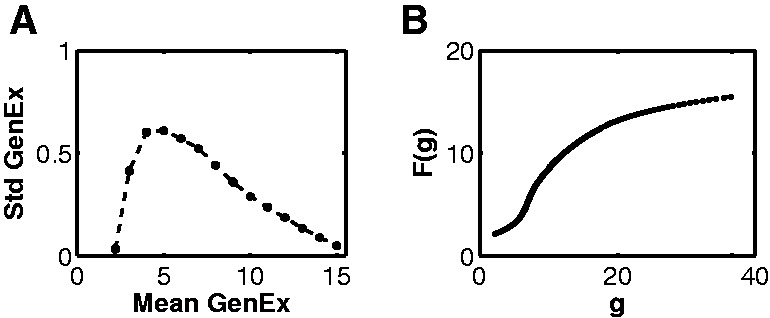
(**A**) Universal noise curve. Standard deviation of gene expression levels over the replicates versus their mean expression level. The mean expression level of replicates for all genes and stimuli was coarse grained into 14 bins, and the standard deviation was averaged for all set of replicates in a given bin. (**B**) Saturation curve of gene expression data, computed by integrating the noise curve. This curve for F was used to remove the saturation effect by applying the inverse of F to the gene expression signal

In this context, the following algorithm was used:

- *Universal noise curve*: The mean and standard deviation was calculated for a given stimulus and gene over the three/four available replicates. The mean expression levels were binned into 14 bins; the mean expression levels within a given bin were combined and their standard deviations averaged. This procedure produces a universal noise curve of the gene expression data, which is shown in Figure 2.
- *Removing the outliers*: For each given gene and stimulus, the replicates were used to calculate the mean expression level. The corresponding noise for that mean expression level was interpolated from the universal noise curve (see above). Replicate measurements that were >3 SD from the mean were designated as outliers and removed. A new mean was calculated using the remaining replicates and the procedure repeated. If only one replicate remained, the gene/stimulus combination was discarded and not used in the subsequent analysis.
- *Linearizing the signal*: The universal noise curve has the peculiar feature that the noise decreases as the mean expression level increases. We attribute this to a saturation effect in microarray signal. One expects that the true measurement noise is independent of the mean expression level. Assuming this, we use the following procedure to correct the observed signal as follows: Let *F*(*g*) be the observed signal (with the saturation non-linearity) when the true signal is *g*. If the noise in *g*, denoted by *dg*, is independent of *g*, then the universal curve is exactly *dF*/*dg*. We can then reconstruct the non-linear filter by simply integrating the noise curve to obtain *F* as a function of *g* and applying the inverse of this function to the mean expression level for each replicate to get a linearized signal (see Figure 2).
- *Signal and its statistical significance*: For the remainder of the analysis, i.e. both training and prediction, the linearized signal was used. We computed the statistical significance of the signal by applying a students *t*-test to the mean expression level of the replicates, after removing outliers as discussed above. This gave us a list of genes that were significantly differentially expressed between the treated/stimulated cells and controls. For the *t*-test, we used the value of the standard deviation from the universal curve corresponding to the computed mean expression level rather than just the standard deviation over the uncorrected replicate measurements.

**Fig. 3.**
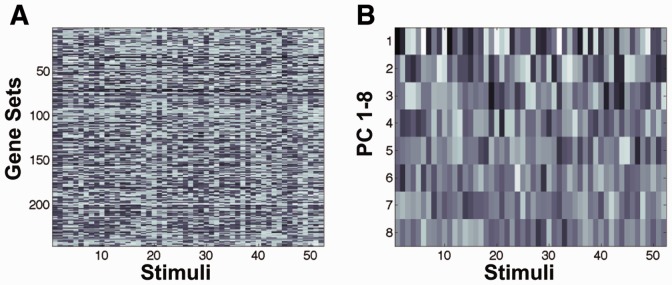
PCA of the training data. (**A**) The gene set enrichment data (NES score) of the 246 rat genes (rows) as a function of the 52 stimuli (columns) used in the experiments. (**B**) The first eight principal components of the data in (A). The first principal component clearly shows the largest variation over the stimuli. The variation decreases for higher ranked components

### 2.3 Processing gene set data

We used no further processing on the GSEA (Subramanian *et al.*, 2005) of the human and the rat genes provided by sbv IMPROVER. The normalized enrichment score (NES) was used as the signal, with the false discovery rate (FDR) value as the corresponding statistical significance for both the human and the rat gene sets. For a complete list of gene sets, see Poussin *et al.* (2014).

### 2.4 Classification algorithm

Principal component analysis (PCA) was performed using Matlab (TM) implementation (pcascat.m, by Marc Strickert), which is publicly available under the GNU public license, version 2, as a part of the package CbMDS at the machine learning open-source software repository: https://mloss.org/software/view/438.

We used *classify* function of the Statistics Toolbox of Matlab (TM) implemented in Matlab R2010b. For the final prediction, *classify* was called using the ‘diaglinear’ option, which fits a multivariate normal density to each group with a pooled estimate of covariance with the non-diagonal entries of the covariance matrix set to zero. This is a naive Bayes classifier (Krzanowski, 1988). We also tried the ‘linear’ option, where the full form of the covariance matrix is used, and saw no improvement. We set the ‘prior’ option of the *classify* routine to ‘empirical’ so that group prior probabilities were estimated from the group relative frequencies in training.

## 3 RESULTS

### 3.1 Designating on/off

A substantial challenge was to find a prudent method for designating when a gene is turned on. Although we had computed the statistical significance of the observed gene expressions for each stimulus (see above), we needed a threshold on the *P*-value to designate a significant expression event. We chose a sharp threshold of *P* < 0.01 to designate a gene expression level as significantly different from that of the control. The accuracy of the prediction did not depend sensitively on this threshold.

For the gene set data, an FDR (Subramanian *et al.*, 2005) < 0.25 was designated as a significant change in expression level in both rat and human. We binarized the gene set data using this threshold.

With *P*-value thresholds for gene expression and gene sets determined, we converted the signals to binary form, with 1 designating a statistically significant change compared with the control and 0 designating no change.

### 3.2 MI of gene sets

In the description below we refer to genes, but the same analysis was also performed with the gene sets. With the binary data at hand, we can define the probability of observing a gene in the on-state over all 26 stimuli. For instance, a gene that is on for 13 of the 26 stimuli has 0.5 probability of being observed in the on-state. We can then compute the Shannon entropy of a given gene using the formula,

(1) $$H\;(c)=-\sum_{c=0,1}p(c)\;\log p(c)$$

Similarly, we can construct a joint distribution for every gene pair in rat and human, which is the probability of observing both the gene in rat and its ortholog in human turning on for a given stimulus. The information content of the joint distribution can be computed in a similar fashion,

(2) $$H\;(g,c)=-\sum_{g=0,1}\sum_{c=0,1}p(g,c)\;\log p(g,c)$$

Finally, the mutual information (MI) for every gene–ortholog pair can be computed from their Shannon entropies and their joint entropy.

(3) I(g,c)=H(c)+H(g)−H(g,c)

A gene–ortholog pair that has high MI is one where the on-/off-state of the rat gene is highly correlated with the state of its ortholog human gene. The expression level of such a rat gene can be useful in predicting the state of its human ortholog. As several human genes are orthologs of the same rat gene and vice versa, we computed gene–ortholog MI for all such pairs, including the multiplicity of ortholog pairs.

**Fig. 4.**
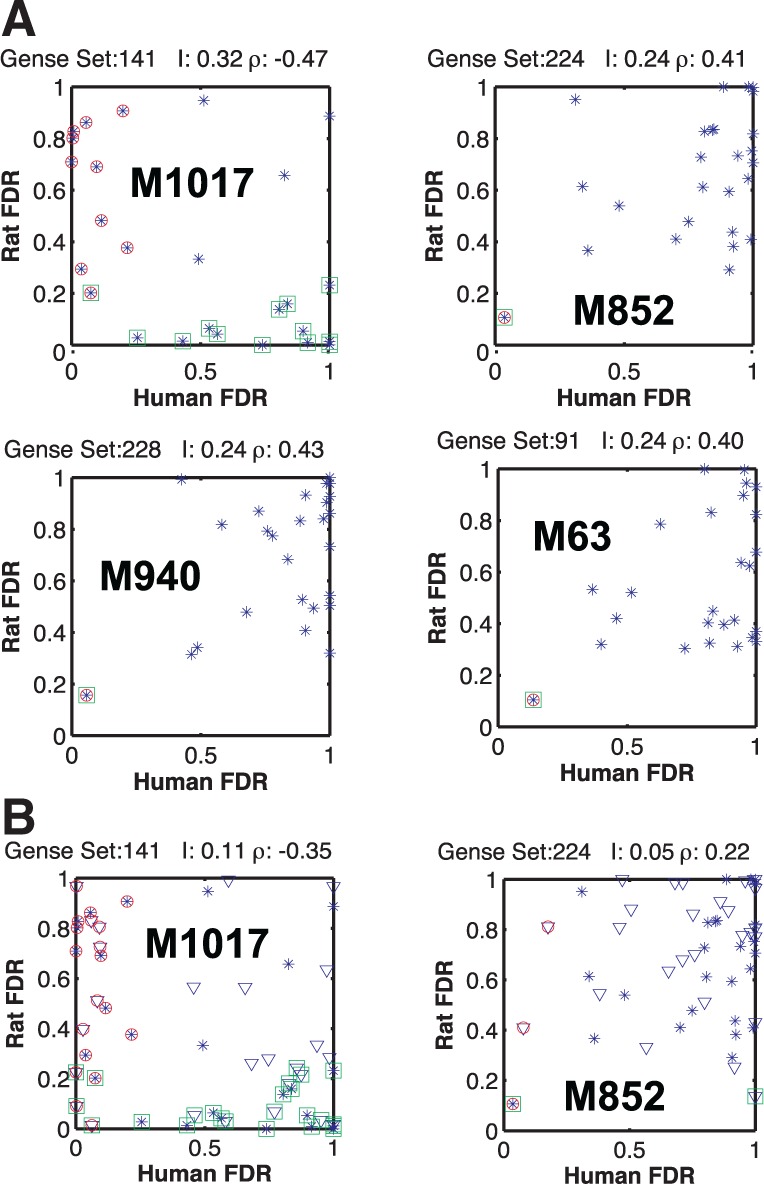
Comparing the correlation between gene sets of human and rat. (**A**) The rat versus human FDR scores of a given gene set are shown for the 26 stimuli in set A (blue dots). Stimuli with expression levels significantly different from those in control are marked with green squares for rat and red circles for human. When the two markers overlap, that particular stimulus results in a similar response in the two species. The highest MI is surprisingly between gene sets that are negatively correlated. In general, the correlation is lower than the intra-species gene and phosphorylation or the inter-species phosphorylation patterns. The legend above each subplot shows the inter-species MI *I* and the Pearson correlation coefficient *ρ*. The gene sets shown are M1017, DNA replication; M852, NF*κ*B activation by TAK1 through phosphorylation and IKKs complex; M940, NF*κ*B activation by TRAF6; M63, osteopontin-mediated events (Subramanian *et al.,* 2005). (**B**) The first two gene sets, with the additional 26 stimuli of the test set also, shown (triangles). M1017 remains the gene set with the highest inter-species MI through anticorrelation. Although MI is lower, the statistical significance is not diminished

Figure 4 shows the top four gene set pairs between human and rat with the highest MI. We observed some interesting features in the inter-species correlations among gene sets. First, the computed inter-species gene set MI and correlation coefficients are low compared with their values in inter-species phosphorylation level (Biehl *et al.*, 2014) and intra-species gene–phospho pairs (Dayarian *et al.*, 2014). A concrete manifestation of this discrepancy can be observed in the cumulative number of gene set activations, where the total number of ON events (FDR < 0.25) for the rat gene sets is three times those of the human gene sets for the 26 known stimuli (set A).

**Fig. 5.**
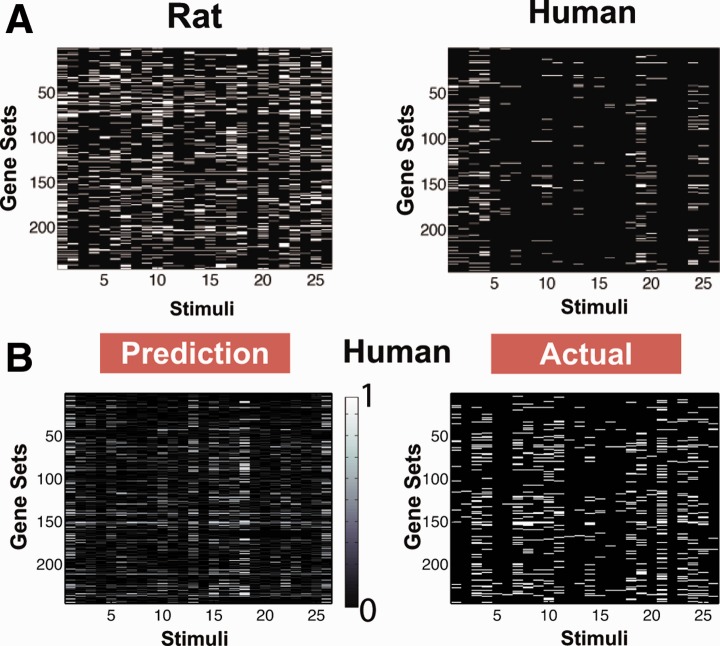
(**A**) Gene set enrichment under the 26 training stimuli for rat and human. The FDR scores of the gene sets are displayed above; white implies differential expression (FDR < 0.25). The number of gene sets that turn on in rat and human are dramatically different. (**B**) The prediction compared with the actual measurements of differential expression in human gene sets under the 26 test stimuli. The color bar indicates level of confidence in the prediction: 0 off, 0.5 uncertain, 1 on

**Fig. 6.**
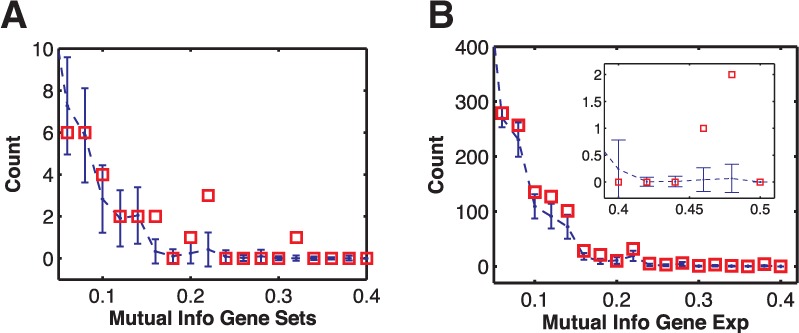
Histogram of the MI for gene set pairs (**A**) and ortholog genes (**B**) in bits. (A) The blue dots with error bars are the mean and standard deviation of counts (of 246 gene sets) obtained from computing MI over randomized datasets. The MI of the actual gene sets at 0.24 and 0.32 (refer to Fig. 4) exceeds the values expected by chance at (*P* < 0.05). (B) Histogram of the MI of ortholog genes (counts are out of 13 841 genes). The ortholog genes do not exhibit a significantly higher MI compared with the gene sets. In fact, because of high number of genes compared with gene sets, it is more likely that high MI in ortholog genes is due to chance

We computed the statistical significance of the MI of the gene sets by repeating the above analysis on randomized datasets obtained by randomly permuting the stimuli. This generates a distribution for MI expected from chance alone, which can be used to infer the *P*-value of the measured MI. The top four gene sets shown in Figure 4 were found to be statistically significant (*P* < 0.05). We find that (Fig. 6A) a MI value < 0.15 bits is no longer statistically significant.

To confirm that the observed correlation is not biased by the choice of the 26 test stimuli, we repeated the same analysis for all 52 stimuli. The MI is lower with all 52 stimuli accounted for; however, the statistical significance does not decrease. This is because it is less likely to observe a high MI by chance for 52 stimuli compared with 26. Moreover, the highest MI gene set remains M1017, and the next three highest remain in the top 5% of the gene sets with highest MI across all 52 stimuli.

We observed the peculiar feature that the gene set with the highest MI (genes involved in DNA replication) was negatively correlated. More precisely, for a given stimulus, when the gene set is activated in human, it is not activated in rat and vice versa (Fig. 4, first panel). To determine whether this is an artifact in the data analysis (possibly owing to the algorithm used for GSEA) or an inherent feature of the data, we computed the correlation between the expression levels of the ortholog genes directly, avoiding the intermediate step of GSEA analysis. Moreover, if the correlation between the expression levels of ortholog genes is significantly larger than that of the gene sets, it is possible to take an indirect route for prediction: predict the gene expression level of the orthologs in Human from that of the Rat, then convert the predicted gene expression pattern to gene set enrichment scores using established algorithms for GSEA. We outline the analysis of the gene expression levels next.

### 3.3 Correlating the inter-species gene expression levels

We considered all rat genes and their corresponding human ortholog (see list of ortholog genes on sbvIMPROVER website). As noted, there was significant redundancy in the ortholog matching, with more than one human gene corresponding to a rat gene and vice versa. For each such ortholog pair, we computed the MI and correlation coefficient over the 26 known stimuli using the method described above. A high MI means that a significant expression of a particular gene in rat under a given stimulus implies significant expression of the ortholog human gene under the same stimulus.

**Fig. 7.**
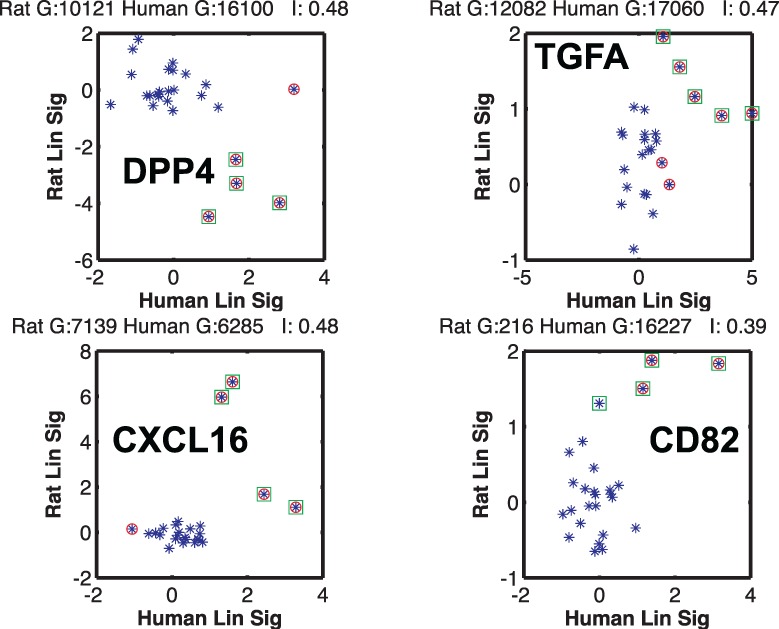
Highly correlated gene orthologs between rat and human. The linearized signal (expression level) of the rat gene is plotted versus its ortholog gene in human for the 26 known stimuli. Marker coloring is same as Figure 4; a significant change in expression in rat is marked with a green square and in human with a red circle. The highest MI occurs for a set of ortholog genes that are negatively correlated. *I* is the MI between the gene orthologs in bits

Figure 7 shows the ortholog pairs with the highest MI. The linearized signal is used to display the expression level of a given gene under a particular stimulus (see Section 2 above). Surprisingly, in agreement with the results from gene set orthologs, the highest MI corresponds to ortholog pairs that are negatively correlated (Rat Gene: 10121 and Human Gene: 16100, DPP4). Here, negative correlation means that when the human gene is over-expressed compared with its control under some stimulus, its rat ortholog is under-expressed compared with its control subject to same stimulus. This suggests that the negative correlation between inter-species gene sets is potentially a consequence of the underlying biology (see Discussion), and not an artifact of the GSEA algorithm.

Furthermore, we computed the correlation coefficient of the expression level of the 192 ortholog genes in the gene set for DNA replication. These genes were on average no more negatively correlated than a randomly chosen pair of ortholog genes. GSEA can go beyond this naive analysis and reveal correlations between groups of genes.

We also considered the statistics of the correlations observed in the ortholog pairs and compared them with the correlations between the gene sets (see histogram in Fig. 6). The ortholog genes seem to exhibit similar levels of inter-species correlation (quantified by MI) compared with the gene sets. However, as many genes correspond to a set, the noise from an indirect comparison might be greater. Also, as there are many more genes than gene sets, the statistical significance of the MI between genes is inevitably lower than that of gene sets. To avoid overfitting in predicting human gene sets in the test data, we decided to use only the gene set enrichment scores.

### 3.4 Predicting the human gene sets

A probabilistic classification algorithm was used to predict the human gene enrichment scores. We used a naive Bayes classifier (Duda *et al.*, 2000; Hastie *et al.*, 2009) implemented using Matlab statistics toolbox (Methods). The training data for the classifier were a simplified representation of the combined rat A and B NES scores using a certain number of principal components (see below for the optimization procedure for the number of principal components). Figure 3 shows the first eight principal components (see Methods for implementation) of rat NES data under the 52 stimuli of sets A and B.

Keeping the leading principal components captures the collective and correlated changes of the gene set under a given stimulus (observed empirically in the data) and discards irrelevant fluctuations from noise. PCA also allows us to consider linear combination of gene sets as opposed to individual gene sets. This should enhance predictive power if the intra-species correlation among gene sets is important. One expects that if gene set A is always differentially expressed when gene set B is on, the state of gene set A can be predicted using the prediction of gene set B, even if a direct prediction of A is not possible from the rat data.

**Fig. 8.**
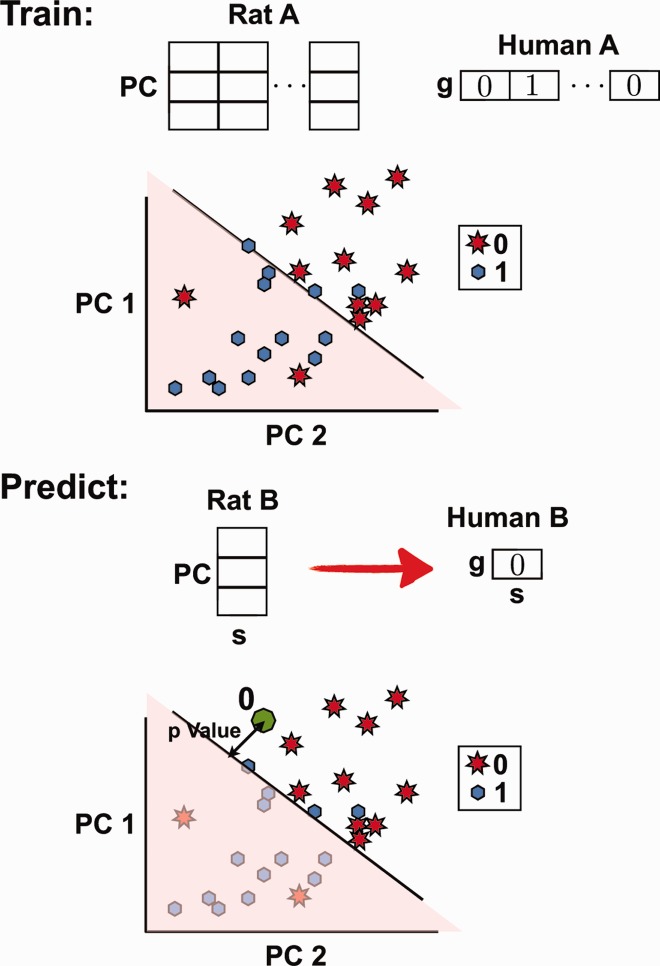
Schematic of the classification protocol. (Top) Training the algorithm to predict gene set *g* in human under stimulus *s*. First, represent the rat NES scores under the 26 stimuli of set A as 26 points in N-dimensional space, where N is the number of principal components used. The figure shows a diagram for *N* = 2. We used *N* = 8 for the actual prediction. Label each of the 26 points as either 0 (off) or 1 (on) based on the human FDR value of gene set *g* under the same 26 stimuli. Next, identify the hyperplane that best separates the two types of labels. (Bottom) Predicting gene set *g* under stimulus *s* of set B. Introduce a new point corresponding to the reduced representation of rat NES score under stimulus s. Depending on which side of the hyperplane the point falls on and its separation distance from the plane, classify as either 0 and 1 and associate a statistical significance

Figure 8 is a diagram for the classifier algorithm. The rat NES scores in sets A and B were combined into a 246 by 52 dimensional matrix, corresponding to the 246 gene sets and the 52 stimuli in sets A and B. PCA was performed to find linear combinations of gene sets that exhibit the largest variation over the 52 stimuli (Fig. 3). For the classification, the N leading principal components (which are linear combination of gene sets) were used. To predict gene set *g* under stimulus *s* (from set B) in human, the following protocol was used. After PCA, the rat NES A data were reduced to 26 points in an N-dimensional space. To each point, we associated the label 1 if gene set *g* was on in human (FDR < 0.25) and the label 0 otherwise. The naive Bayes classifier was used to find a hyperplane that separated the 0 s from the 1 s. In general, an error-free linear separation cannot be achieved. To make the prediction, the rat NES score under stimulus *s* was expressed in terms of the principal components and added as new point into the N-dimensional space. Depending on which side of the hyperplane the point falls on, a classification of 0 or 1 was assigned.

The pseudo-code of the above algorithm with an additional leave-one-out verification step is as follows:


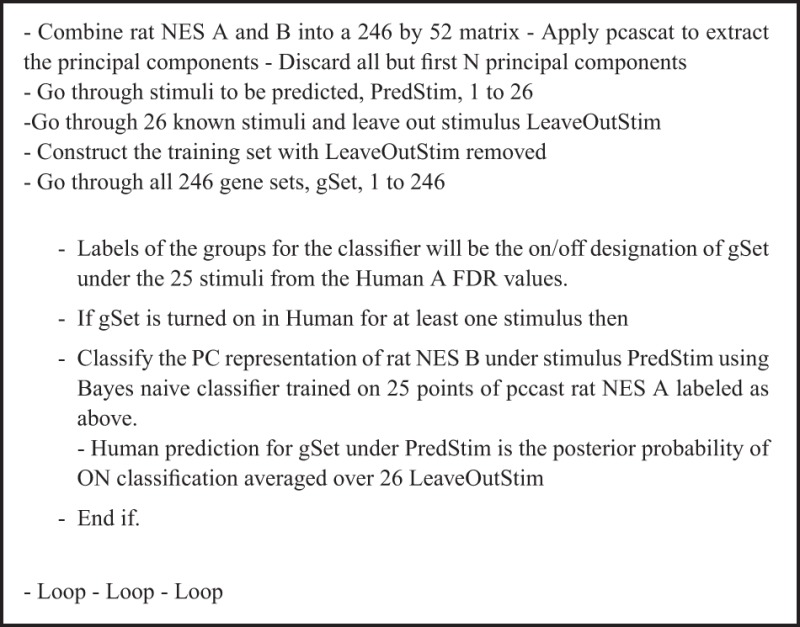


The Matlab (TM) *classify* function provides a posterior probability of the input belonging to a certain classification group. We used the probability of the gene being designated ON as our prediction. Moreover, instead of training on the 26 known stimuli, we averaged 26 classifiers constructed from recursively leaving out one of the known stimuli.

**Fig. 9.**
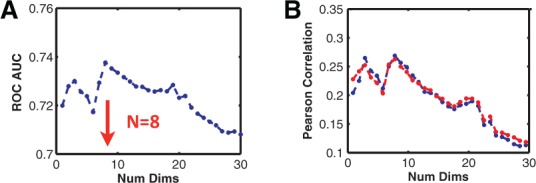
Optimizing the number of principal components used for prediction. The classifier used was a naive Bayes classifier, applied to 25 of the 26 known stimuli (A set) to predict the 26th. All metrics: area under the ROC curve (**A**), Pearson correlation coefficient (B) and Matthews correlation coefficient (not shown) are maximum near *N* = 8, suggesting that 8 leading principal components are optimal. (**B**) The blue line corresponds to correlating the prediction to the binarized known FDR values (ON when FDR < 0.25) and the red line to correlation with the FDR values converted to a continuous scale (using 1-FDR value)

An important free parameter in the above algorithm is the number of principal components used in the prediction. To optimize this, we applied the algorithm to the 26 known stimuli (set A) by predicting one of the stimuli from training on the remaining 25. The output of the classifier was then compared with the actual measurement. Various metrics such as the area under the receiver-operating characteristic (ROC) curve (Davis and Goadrich, 2006; Fawcett, 2006), Pearson correlation coefficient and Matthews correlation coefficient were used to quantify the performance of the classifier (see Fig. 9). The classifier was optimized when eight leading principal components were used. We also repeated the analysis using a linear discriminant analysis algorithm (Methods); the performance did not improve significantly. For the final prediction, we used *N* = 8 and applied a leave-one-out procedure to the naive Bayes classifier.

Our final prediction achieved the best performance among all seven teams that participated in this sub-chellenge. Table 1 shows that our approach achieved the highest score for area under precision-recall (AUPR) and Pearson Correlation, as well as tying for first with two other teams in the balanced accuracy (BAC). Teams were ranked according to their sum of ranks over these three metrics. The organizers performed a robustness analysis of the rankings by sampling 10% of the gold standard against which to rescore the teams. This was performed 1000 times, and samples were drawn in such a way as to maintain the same proportion of positive and negative classes as in the complete gold standard. This analysis shows that our method’s high performance is robust to the composition of the gold standard, and it ranks consistently higher than the other approaches (Rhrissorrakrai *et al.*, 2015).

**Table 1.** Comparing performance of various predictions to measurements

Team	AUPR	Pearson	BAC	Rank
Team 50*	0.19	0.59	0.54	1
Team 133	0.12	0.54	0.54	2
Team 49	0.12	0.53	0.53	3
Team 52	0.10	0.52	0.54	4
Team 131	0.11	0.50	0.52	5
Team 105	0.11	0.52	0.51	6
Team 111	0.06	0.41	0.43	7

The algorithm described in this article is that of Team 50. The predicted response to the 26 stimuli of set B is compared with the measured response using the AUPR, Pearson correlation coefficient and BAC. For details on these metrics and the scoring see (Rhrissorrakrai *et al.*, 2015). Asterisk denotes the author's team number in the challenge.

## 4 DISCUSSION

We devised an algorithm to predict differential gene set enrichment in NHBE primary cells under various stimuli from measurements of NRBE primary cells exposed to the same stimuli. As a part of our analysis, we computed the MI between the differential enrichment of the corresponding gene sets and also ortholog pairs of genes in the two species. MI objectively quantifies the predictive power of the rat data without a need to assume an underlying model. It also determines the level of difficulty of the prediction; higher MI naively implies that inference should be easier.

We found that 6 h after exposure, gene set enrichment in NHBE is significantly different than in NRBE, despite almost identical response in protein phosphorylation states measured 5 and 25 min after exposure. As phosphorylation happens close to the beginning of the signaling cascade, it is expected that the response at a fixed early time point should be more conserved between the two species. As the signal is transmitted further along the signaling pathway, slight differences between the two species can result in divergence of their response at later time points. This is principally manifested in the inverse relationship observed between the human and rat gene set that had the highest MI (DNA replication gene set). To rule out a bias from GSEA analysis as the cause, we computed the MI directly for each pair of ortholog genes. The ortholog gene pair with the highest MI (DPP4) was also negatively correlated in expression levels between the two species.

How can a gene set be differentially expressed in the NHBE cells when they are not in rat cells (and vice versa) under the same stimuli? One possibility is that this reflects an evolutionary divergence of the particular pathway in the two species. However, this is unlikely, as it would imply that biological modules are completely anticorrelated in function in NHBE cells compared with NRBE cells. A more likely explanation is that the discrepancy stems from a time difference between rat and human in how the phosphorprotein activates downstream genes. It reflects the limitation of the measurement process used in this challenge, where gene expression levels were measured at the arbitrary time point of 6 h in both species. It is conceivable that the pathways exhibit non-trivial dynamics following activation/inactivation by the same stimulus. During the response, some genes might oscillate or transiently be under-expressed following a spike in expression levels (Levine *et al.*, 2013). In fact, DNA replication genes are normally tuned to the cell cycle; related pathways such as p53 are known to exhibit such oscillation (Bar-Or *et al.*, 2000; Lahav, 2008). If the phase or the period of these oscillations is different in rat and human, a snapshot measurement can potentially exhibit anticorrelations. This would explain why we find that anticorrelated pathways have the highest MI because they are the ones that are oscillating after being triggered by the stimulus. To validate and further understand the role of such dynamics, the microarray analysis would need to be repeated for a number of time points following stimulation. A time series of the gene expression levels would then elucidate the differences between the dynamics of the response in the two species, shedding more light on the underlying biology.

Despite the low MI between the gene sets of the two species, we were able to make non-trivial predictions of the response of NHBE cells based on measurements in NRBE cells, which were verified experimentally (Rhrissorrakrai *et al.,* 2015). The inference algorithm relied only on the state of the gene sets. We found that the individual ortholog genes did not exhibit significantly higher MI. It is plausible that biological modules and pathways are better conserved across the two species whereas expression levels of individual genes are not. More measurements are needed to verify this claim. Predicting coarse-grained modules at various levels of hierarchy could be more biologically relevant for species translation, as opposed to predicting expression levels of individual genes or post-translational state of individual proteins.

We relied heavily on PCA of the gene sets to make the inference. PCA served two broad purposes. First, it allowed us to eliminate noise in the gene set enrichment scores. More importantly, it enabled us to naturally account for intra-species correlations between gene sets. By linearly combining the gene sets, we could predict enrichment of gene sets in human that had almost no correlation with their counterpart in the rat but were well-correlated with other human gene sets. We did not explicitly consider possible correlations in the response between different stimuli from their underlying biology, as the inference was made with no knowledge of the test stimuli.

Inter-species inference of gene expression is potentially more difficult than inter-species inference of protein phosphorylation states (Biehl *et al.*, 2014), and intra-species prediction of gene expression from protein activation (Dayarian *et al.*, 2014). Nevertheless, the methods described here could infer differential gene set enrichment in human epithelial lung cells from proteomic and transcriptomic data on rat epithelial cells, with reasonable accuracy. Such a framework is potentially useful to translate measurements in rats to humans, with therapeutic applications in drug development, or for understanding basic biological mechanisms.

## References

[btu569-B1] Bar-Or L (2000). Generation of oscillations by the p53-mdm2 feedback loop: a theoretical and experimental study. Proc. Natl Acad. Sci. USA.

[btu569-B2] Biehl M (2014). Inter-species prediction of protein phosphorylation in the sbv IMPROVER species translation challenge. Bioinformatics.

[btu569-B3] Davis J, Goadrich M (2006). The relationship between Precision-Recall and ROC curves. Proceedings of the 23rd International Conference on Machine learning (ICML ’06).

[btu569-B4] Dayarian A (2014). Sbv Improver sub-challenge 1: learning and predicting phosphorylation levels of upstream effectors in rat lung epithelial cells. Bioinformatics.

[btu569-B5] Duda RO, Hart PE, Stork DG (2000).

[btu569-B6] Fawcett T (2006). An introduction to ROC analysis. Pattern Recogn. Lett..

[btu569-B7] Hackam DG, Redelmeier DA (2006). Translation of research evidence from animals to humans. JAMA.

[btu569-B8] Hastie T (2009). The Elements of Statistical Learning: Data Mining, Inference, and Prediction.

[btu569-B9] Krzanowski WJ (1988). Principles of Multivariate Analysis: A User’s Perspective.

[btu569-B10] Lahav G (2008). Oscillations by the p53-Mdm2 feedback loop. Adv. Exp. Med. Biol..

[btu569-B11] Levine JH (2013). Functional roles of pulsing in genetic circuits. Science.

[btu569-B12] Mestas J, Hughes CC (2004). Of mice and not men: differences between mouse and human immunology. J. Immunol..

[btu569-B13] Meyer P (2012). Industrial methodology for process verification in research (IMPROVER): toward systems biology verification. Bioinformatics.

[btu569-B14] Pound P (2004). Where is the evidence that animal research benefits humans?. BMJ.

[btu569-B15] Poussin C (2014). The species translation challenge a systems biology perspective on human and rat bronchial epithelial cells. Scientific Data.

[btu569-B16] Rhrissorrakrai K (2015). Understanding the limits of animal models as predictors of human biology: lessons learned from the sbv IMPROVER Species Translation Challenge. Bioinformatics.

[btu569-B17] Rice J (2012). Animal models: not close enough. Nature.

[btu569-B18] Sato N (2004). Maintenance of pluripotency in human and mouse embryonic stem cells through activation of Wnt signaling by a pharmacological GSK-3-specific inhibitor. Nat. Med.

[btu569-B19] Seok J (2013). Inflammation and Host Response to Injury, Large Scale Collaborative Research Program. Genomic responses in mouse models poorly mimic human inflammatory diseases. Proc. Natl Acad. Sci. USA.

[btu569-B20] Subramanian A (2005). Gene set enrichment analysis: a knowledge-based approach for interpreting genome-wide expression profiles. Proc. Natl Acad. Sci. USA.

[btu569-B21] Tu Y (2002). Quantitative noise analysis for gene expression microarray experiments. Proc. Natl Acad. Sci. USA.

[btu569-B22] van der Worp HB (2010). Can animal models of disease reliably inform human studies?. PLoS Med..

[btu569-B23] Wu Z (2004). A model-based background adjustment for oligonucleotide expression arrays. J. Am. Stat. Assoc..

